# Ovarian Carcinoid Misinterpreted as Endometrioid Adenocarcinoma in Mature Cystic Teratoma

**DOI:** 10.7759/cureus.11948

**Published:** 2020-12-07

**Authors:** Prerna Tewari, Jitendra S Nigam, Tarun Kumar, Avinash Singh, Jagjit Pandey

**Affiliations:** 1 Pathology and Laboratory Medicine, All India Institute of Medical Sciences, Patna, Patna, IND; 2 Pathology, All India Institute of Medical Sciences, Patna, Patna, IND; 3 Surgical Oncology, All India Institute of Medical Sciences, Patna, Patna, IND

**Keywords:** ovary, carcinoid, endometrioid carcinoma, teratoma

## Abstract

Mature cystic teratoma (MCT) is the most common benign germ cell tumor of the ovary and contains the different tissues that originate from the endoderm, mesoderm, and ectoderm. The monodermal teratoma has a component of only the germ layer. Ovarian carcinoid is rare and considered as a monodermal teratoma. We report a case of carcinoid tumor arising in MCT in a 60-year-old postmenopausal woman.

## Introduction

Mature cystic teratoma (MCT) is the most common benign germ cell tumor of the ovary, and 1% - 3% of cases may undergo malignant transformation [1-4]. The tumors emerging in MCT are commonly squamous cell carcinoma, adenocarcinoma, and less commonly carcinoid [1-3]. The ovarian carcinoid tumors constitute approximately 0.3% of all carcinoids and <0.1% of all ovarian cancers [1,3]. We report an uncommon case of carcinoid tumor arising in MCT in a 60-year-old postmenopausal woman.

## Case presentation

**Figure 1:**
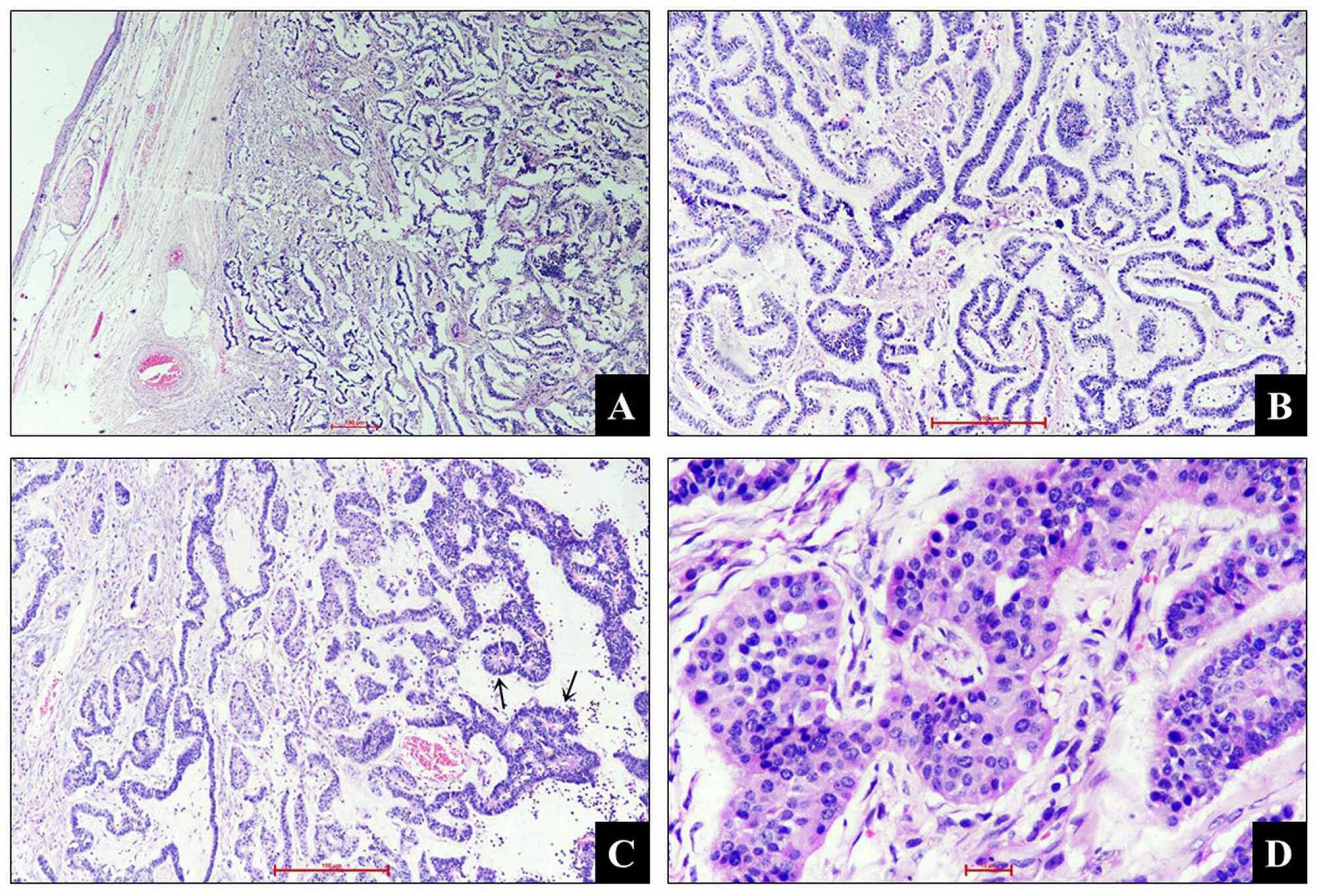
Hematoxylin and eosin stain A. An ectodermal component consists of strips of stratified squamous epithelium along with the sebaceous gland and monomorphic tumor cells. (H&E x40); B& C. Tumour cells arranged in insular, trabecular, and anastomosing cords pattern. (H&E x100); D. Round to oval uniform nuclei with stippled nuclear chromatin and a moderate amount of eosinophilic granular cytoplasm. (H&E x400)

A 60-year-old female visited a private hospital with complaints of postmenopausal bleeding for two months. A complex cystic lesion of size 11.3x8.6x5.7 cm in the hypogastrium was observed by ultrasonography. A diagnosis of an ovarian dermoid rendered by radiological evaluation. Serological tumor markers were within normal limits. The patient undergoes laparotomy, with right salpingo-oophorectomy. A histopathological diagnosis of endometrioid adenocarcinoma arising in MCT was rendered outside. The patient was referred to our institute, and we reviewed her corresponding slides and blocks. The sections reviewed showed tumor cells arranged in insular, trabecular, and anastomosing cords pattern. These tumor cells were round, predominantly uniform nuclei with stippled nuclear chromatin and had a moderate amount of eosinophilic granular cytoplasm. Strips of stratified squamous epithelium along with the sebaceous unit were also identified (Figure 1).

**Figure 2:**
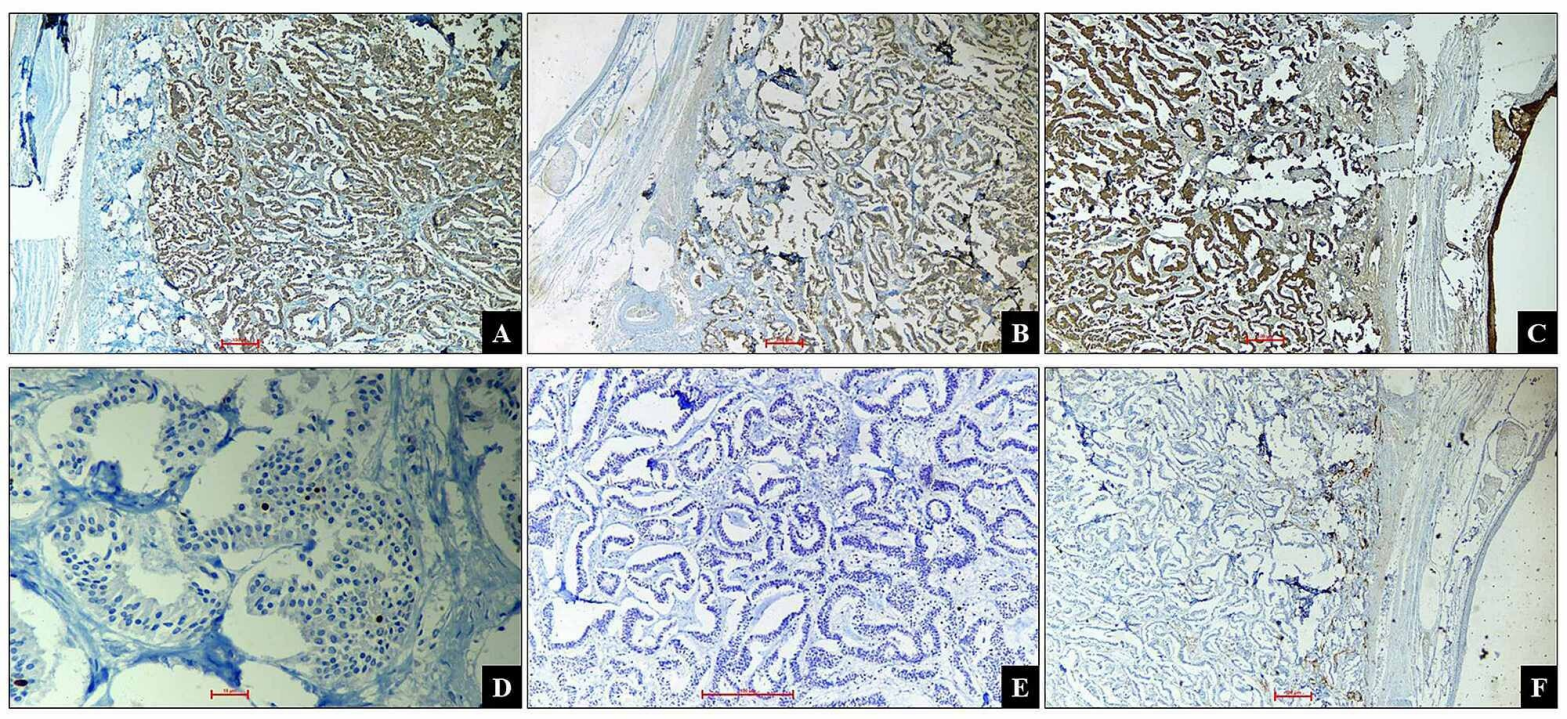
Immunohistochemistry A. Chromogranin: Strong and diffuse cytoplasmic positivity in tumor cells. (x40); B. Synaptophysin: Strong and diffuse cytoplasmic positivity in tumor cells. (x40); C. Pancytokeratin: Strong and diffuse cytoplasmic positivity in tumor cells. (x40); D. Ki67: <1% nuclear positivity. (x400); E. Estrogen Receptor: negative in tumor cells. (x100); F. Inhibin: Negative in tumor cells. (x40)

No necrosis or increase in mitotic activity was seen. No other teratomatous components were observed. On immunohistochemistry (IHC), the tumor cells showed strong, diffuse, cytoplasmic positivity for chromogranin and synaptophysin. The pan-cytokeratin showed strong, diffuse, cytoplasmic, and membranous positivity. The tumor cells were negative for Inhibin and Estrogen receptor (ER). Ki-67 was less than 1% (Figure 2).

Histomorphological and IHC findings favored the diagnosis of ovarian carcinoid arising in MCT (possibly monodermal teratoma).

## Discussion

Teratoma contains the different tissues from the three germ cell layers, the endoderm, mesoderm, and ectoderm [3]. The ovarian carcinoid is a rare but second most common monodermal teratoma after struma ovarii [5]. They are usually seen in perimenopausal or early postmenopausal females [5]. More than 50% of ovarian carcinoids reported as a component of MCT [6]. These patients may present with an enlarging abdominal or pelvic mass [1-6]. The other uncommon symptoms include constipation, urinary frequency, abdominal pain, hirsutism may also be present [1-6]. The symptoms like cutaneous or facial flushing, bronchospasm, abdominal cramps, diarrhea, edema, carcinoid heart disease, etc., are also reported in these patients [5,6]. These signs and symptoms are related to bioactive mediators secreted by carcinoid tumor cells [5,6]. The radiologic identification of fat, cartilage, teeth, hair, and bone in the cystic lesion helps diagnose the cystic teratoma preoperatively [1,3]. However, MCT's malignant transformation is not straightforward on radiology and requires histopathological evaluation for the conclusive interpretation [1,3]. The indexed case was presented with postmenopausal bleeding with evidence of complex cystic ovarian dermoid on radiology. Based on the histomorphologic features, ovarian carcinoids are classified into five subtypes: insular, trabecular, mucinous, mixed, and strumal carcinoid [1-6]. The insular carcinoid is the most common type and commonly associated with carcinoid syndrome [3,5]. However, trabecular carcinoids are the second most common type and have a good prognosis [1,3]. Like the neuroendocrine neoplasm of other sites, ovarian carcinoids also express ≥1 neuroendocrine [1-6]. IHC markers like synaptophysin, chromogranin, or CD56 with variable immunoreactivity for pan-cytokeratin and keratin 7 [1-6]. The expression of CDX2 was also reported in ovarian insular and mucinous carcinoids and restrained its use to exclude the gastrointestinal carcinoid metastasis to the ovary [2,5]. The primary carcinoid of the ovary is considered only after eliminating the metastatic carcinoid [5]. The presence of extra ovarian carcinoid, bilaterality, multinodular growth, history of carcinoid neoplasm at other sites, and persistence of signs and symptoms of carcinoid syndrome after surgical excision of tumor supports the metastatic disease [5]. However, the unilaterality and associated other primary ovarian neoplasm favor an ovarian origin [5]. Surgical management is considered as the initial approach [1,3]. In a postmenopausal patient with malignant transformation in MCT, radical hysterectomy may be contemplated. The local excision may be the best treatment approach for young and nulliparous patients that require the preservation of fertility [1,3]. However, most cases have been diagnosed postoperatively [6]. The primary ovarian carcinoid limited to the ovary has approximately 100% ten years survival rates, and in the advanced stage, it decreases to 33% for the five years survival rate [3]. In the present case, the right salpingo-oophorectomy was done with an uneventful postoperative course for two months.

## Conclusions

Primary ovarian carcinoid is a rare entity and can have deceptive histology, which may lead to misdiagnosis. Thereby, IHC is necessary to confirm the diagnosis along with the exclusion of secondary carcinoid. Since the preoperative diagnosis of malignant transformation in MCT is not easy, extensive sampling must not miss a focus of malignant transformation.
